# Un siglo de asistencia en el Hospício Nacional de Alienados do Rio de Janeiro, 1852-1944

**DOI:** 10.1590/S0104-59702024000100034

**Published:** 2024-07-15

**Authors:** Olga Villasante

**Affiliations:** i Psiquiatra, Hospital Universitario Severo Ochoa. Madrid – Espanha olga.villasante@salud.madrid.org

La historia de los establecimientos psiquiátricos, como espacios dedicados a la observación y tratamiento de enfermedades mentales, ha ocupado numerosas páginas de la historiografía de la medicina mental en las últimas décadas. Entre las instituciones brasileñas, el Hospício de Pedro II – Hospício Nacional de Alienados (HNA), fundado en 1852 y objeto del libro que nos ocupa, ha sido epicentro de diversos proyectos de investigación como el desarrollado entre 2015 e 2018 en el Departamento de Pesquisa em História das Ciências e da Saúde da Casa de Oswaldo Cruz, financiado por el Programa de Excelência em Pesquisa/Fundação Oswaldo Cruz/Conselho Nacional de Desenvolvimento Científico e Tecnológico y coordinado por Cristiana Facchinetti (2022). En este libro la profesora Ana Teresa A. Venancio y el investigador Allister A. Teixeira Dias, que ya habían publicado numerosos trabajos sobre esta institución, coordinaron a un vasto grupo de investigadoras que incluyen sociólogas, psicólogas, documentalistas etcétera, para generar un amplio *corpus* de conocimiento sobre el Hospício Nacional de Alienados. La obra, fruto del proyecto de investigación “Do Hospício de Pedro II ao Hospício Nacional de Alienados: cem anos de histórias (1841-1944)”, y que tejen con pericia y buen hacer Ana Teresa y Allister, aborda diferentes momentos de la historia brasileña, comenzando por el último monarca del Imperio brasileño, marcado por intereses esclavistas, para finalizar en la era de Getúlio Vargas (1930-1945).

El libro está estructurado en cinco partes, precedidas por el prefacio, a cargo del profesor Rafael Huertas y la presentación de Venancio y Teixeira. En la primera parte ya queda claro que las fuentes dominan la investigación, en palabras de Huertas (2001), cumpliendo uno de los objetivos del libro. Sin duda, esta investigación fomenta la preservación del acervo del HNA que pertenece a cuatro instituciones: Instituto Municipal de Assistência à Saúde Nise da Silveira, Instituto Municipal de Assistência à Saúde Juliano Moreira, Instituto de Psiquiatria da Universidade Federal do Rio de Janeiro y Museu Penitenciário do Estado do Rio Janeiro. Para empezar, Laurinda Rosa Maciel, Daniele Corrêa Ribeiro y Cátia Maria Mathias reflexionan sobre el valor del patrimonio cultural de los citados archivos, además de otros como el Arquivo Nacional o el Arquivo Geral da Cidade do Rio de Janeiro. El elevado potencial de este magnífico trabajo archivístico se debe a la sistematización y accesibilidad del ingente *corpus* documental (libros de observación clínica, fotografías) que resultan ser un faro para bibliotecarios e investigadores, no solo noveles. Facchinetti y Teixeira plantean una interesante reflexión teórico-metodológica en la que analizan la influencia de los postulados foucaultianos en torno a la psiquiatría como disciplina de poder y de la perspectiva sociológica de Roy Porter, Andrew Scull o George Rosen. Además, se destacan las razones para la utilización de fuentes clínicas que introducen una polifonía de registros (historiales médicos, cartas), implicando abordajes en clave “asimétrica” como el planteado por Rivera Garza. El investigador senior Carlos Estellita-Lins cierra este apartado, resaltando el valor de la semiología psiquiátrica y neurológica en los primeros 45 años del siglo XX. Argumenta, no obstante, que los aspectos neurológicos se perciben más que los psiquiátricos en las sesiones y los casos publicados; sin duda, derivados de la influencia de los manuales y libros en la práctica clínica de los médicos.

La segunda parte, centrada en la época imperial, la abre Flavio Coelho Edler, que realiza una revisión de *Danação da norma*, la obra seminal de Machado et al. (1978). Se aportan argumentos para considerar el asilo como una respuesta específica del poder público a un problema de orden urbana y se cuestiona cómo el uso polisémico del concepto de medicalización permite explorar distintos significados del espacio asilar. Posteriormente, Daniele Corrêa Ribeiro recopila los registros de entrada en el hospicio definiendo quienes eran las instituciones que derivaban pacientes entre 1850 y 1889. El principal valor del trabajo es la observación del flujo de pacientes y el análisis sobre otras categorías, al modo expuesto por Ian Hacking, que influyeran en el ingreso, además de las categorías médicas. Especialmente interesante es el trabajo de Michelly Vieira da Silva y María Renilda Barreto, quienes exploran los ingresos de la población negra, procedente de la esclavización de africanos (Moçambique, Benguela, Cabinda, Mofundi). A partir de los datos, las investigadoras reflexionan sobre los factores que contribuyeron a una mayor exclusión de la población negra (ausencia de *locus* social) que, fundamentalmente, ocupaban la tercera clase en el hospicio. El último capítulo de esta parte lo desarrolla Ana María G.R. Oda, profesora de la Universidade Estadual de Campinas, que estudia con maestría la manía, monomanía y demencia, diagnósticos más comunes entre 1870 y 1880. Las posibles diferencias en su utilización por médicos como Nuno Ferreira de Andrade o Teixeira Brandão nos brinda una oportunidad para reflexionar sobre la historia conceptual de la psiquiatría, la recepción de las categorías clínicas o la relaciones entre cuerpo, mente y moral.

En la tercera parte se aborda la Primera República (1989-1930), un periodo marcado por la modernidad y el progreso, que trató de instaurar una asistencia “más científica”, abandonando aspectos más custodiales. Se comienza por el análisis del Pavilhão de Observação, de Cátia Maria Mathias, cuando la institución es transferida a la Universidade do Brasil, denominándose Instituto de Psiquiatría. El trabajo huye de la vieja historia institucional, reconstruyendo las conexiones entre asistencia y los cambios legislativos que influyeron en el quehacer psiquiátrico cotidiano. Un segundo texto, cuya autora es Mónica Cristina de Moraes, aborda el tratamiento de los tuberculosos en el Hospicio, aislados en los pabellones De Simoni y Sigaud. La autora no se conforma con realizar una descripción de los pabellones que acompaña con fotografías, sino que se preocupa por la función del aislamiento en una enfermedad, cuyos condicionantes socio-higiénicos están fuera de toda duda. Cierra este tercer apartado el trabajo de Allister A. Teixeira Dias (2017), quien discute la relación histórica entre crimen y locura partir de la Seção Lombroso del HNA, un pabellón reservado a “locos criminales”. Este trabajo aporta una interesante discusión sobre aquellos individuos “doblemente desgraciados”, en palabras de Heitor Carrilho, que fueron el centro de un debate sobre las ideas regeneracionistas filtrado y discutido desde el paradigma médico/naturalístico y el jurídico.

La cuarta parte se inaugura con una investigación a cargo de un grupo de psicólogas, liderado por Ana María Jacó Vilela, quienes detallan los test utilizados en el Gabinete de Psicología entre 1907 y 1925. Un abordaje preliminar que abre, no obstante, un campo de estudio sobre el uso práctico de los tests en el HNA de utilización cada vez más frecuente en dichas décadas. Este trabajo es seguido del de Pedro Muñoz, que explora la trayectoria de Ulysses Vianna Filho y su labor en el Laboratorio Nissl de la Seção Pinel del HNA, donde trató de desentrañar el sustrato biológico de la locura. Se nos proporcionan claves para conocer la influencia germana a través de este médico pernambucano que viajó con frecuencia a Alemania, estableciendo un intercambio científico, en un momento en el que se debatía la separación de la neuropsiquiatría en dos especialidades diferenciadas. En el siguiente capítulo, firmado por la experta investigadora Ana Teresa A. Venancio y por José Roberto Saiol, se estudia la retirada de Juliano Moreira del HNA en 1930 (Venancio, Potengy, 2015). Dado el silencio del médico, se trata de explorar, a través de la prensa, los intereses y la coyuntura de la salida de Moreira que marca una nueva era, al finalizar la reivindicación republicana. Para concluir este bloque Yonissa Marmitt Wadi relata el caso de Karina, donde las palabras de ella toman un protagonismo poco habitual en la historiografía. El acercamiento a la transformación de una mujer común en una loca, a partir de sus escritos y bordeando el discurso del experto (médico), está inspirado en Foucault o Rivera-Garza.

La quinta parte está constituida por un único trabajo con una detallada cronología que relaciona la asistencia psiquiátrica a los alienados desde 1808 hasta 1944, con la historia de la salud y de la psiquiatría brasileña. Paralelamente, se enmarcan históricamente los acontecimientos más relevantes del contexto nacional e internacional, constituyendo una magnifica herramienta para quien quiera acercarse a la exploración de otras áreas de investigación u otros periodos de la historia de la locura brasileña.

En definitiva, este libro coordinado por Ana Teresa A. Venancio y Allister Teixeira Dias (2022) nos imbuye de una reflexión historiográfica que abarca la historia institucional, la historia de los saberes y las prácticas psiquiátricas, pero también la historia de personas, no solo del personal sanitario, sino de pacientes con una historia hecha “desde abajo” que abre la mirada de la subjetividad.

No podemos finalizar sin señalar que este volumen no solo puede interesar al público especializado en antropología, sociología, historia de la psicología o psiquiatría, entre otras disciplinas, sino también es idóneo para un público universitario más joven. El conjunto resulta muy rico en abordajes de modo que su lectura da claves para la utilización de los archivos y el manejo de fuentes y bibliografía secundaria a aquellas personas que se adentren en sus primeras investigaciones, tesis de maestría o doctorado, una cualidad que se debe a su coordinadora y coordinador.
